# Characterizing the Cellular Constituents of Proximal Airway Disease in Granulomatosis With Polyangiitis

**DOI:** 10.1002/ohn.1197

**Published:** 2025-03-10

**Authors:** Wenda Ye, Evan Clark, Edward Talatala, Ruth Davis, Marisol Ramirez‐Solano, Quanhu Sheng, Jing Yang, Sam Collins, Alexander Hillel, Alexander Gelbard

**Affiliations:** ^1^ Department of Otolaryngology–Head and Neck Surgery Vanderbilt University Medical Center Nashville Tennessee USA; ^2^ Department of Otolaryngology–Head and Neck Surgery University of Wisconsin School of Medicine and Public Health Madison Wisconsin USA; ^3^ Department of Biostatistics Vanderbilt University Medical Center Nashville Tennessee USA; ^4^ Department of Otolaryngology–Head and Neck Surgery Johns Hopkins University Baltimore Maryland USA

**Keywords:** airway fibrosis, granulomatosis with polyangiitis, immunology, single‐cell RNA sequencing, subglottic stenosis

## Abstract

**Objective:**

Granulomatosis with polyangiitis (GPA) is a rare multisystem autoimmune vasculitis. 10‐20% of patients suffer life‐threatening obstruction of their proximal airways. Although progress has been made in the treatment of systemic disease, ameliorating airway disease in GPA remains an unmet need arising from limited understanding of disease pathogenesis. We sought to characterize the cellular constituents of the affected proximal airway mucosa in GPA airway scar.

**Study Design:**

Basic/translational study.

**Setting:**

Single tertiary care center.

**Methods:**

Using single‐cell RNA sequencing, we profiled the cellular constituents of proximal airway samples from GPA and disease comparators (GPA; n = 9, idiopathic subglottic stenosis: iSGS; n = 7, post‐intubation proximal stenosis: PIPS; n = 5, and control; n = 10). We report transcriptomes for subglottic epithelial, immune, endothelial, and stromal cell types and map expression of GPA risk genes to tissue types present in the proximal airway. We compared differential gene expression across immune cell populations and performed pseudotime analysis using Monocle 3.

**Results:**

Similar to iSGS and PIPS, the subglottic mucosa of GPA patients demonstrated an abundant immune infiltrate. 71% of the established GPA risk genes (10 of 14) localized to T cells and macrophages. Differential gene expression and pseudotime analysis revealed a sub‐population of CD4‐/CD8‐ inflammatory T cells that only originated from GPA.

**Conclusion:**

We characterized the cellular composition of GPA airway disease and demonstrated that the expression of GPA risk alleles is predominantly localized to immune cell populations. We also identified a subset of inflammatory T cells that is unique to GPA.

Granulomatosis with polyangiitis (GPA, formerly “Wegener's granulomatosis”) is rare, systemic small‐vessel vasculitis characterized by necrotizing granulomatous inflammation that commonly involves the lungs, kidneys, and head/neck (Figure 1A‐C).^1^, ^2^, ^3^ In particular, disease in the head and neck region has been shown to affect up to 92% of patients with GPA and may manifest as sinonasal, laryngotracheal, and/or otologic symptoms.^3^, ^4^, ^5^ Of these, upper airway obstruction may develop into a serious life‐threatening complication, with subglottic disease involvement present in as many as 16% of patients with GPA.^6^ Although the precise etiology of the disease is unknown, immune dysregulation and autoimmunity are heavily implicated, with the characteristic presence of antineutrophil cytoplasmic antibodies (ANCA) against proteinase 3 (PR3) in patients with GPA.^5^ As a result, treatment algorithms have traditionally revolved around systemic immunosuppression with agents such as cyclophosphamide, and more recently, rituximab.^2^, ^7^, ^8^, ^9^ Prior to the 1970s, patients with GPA had a 1‐year mortality rate of >80%, primarily due to renal or lung failure. The introduction of combination cyclophosphamide and glucocorticoid treatment significantly reduced mortality, turning GPA into a chronic disease.^10^ Use of the targeted biological therapy rituximab has continued to improve survival and reduce toxicity.

**Figure 1:**
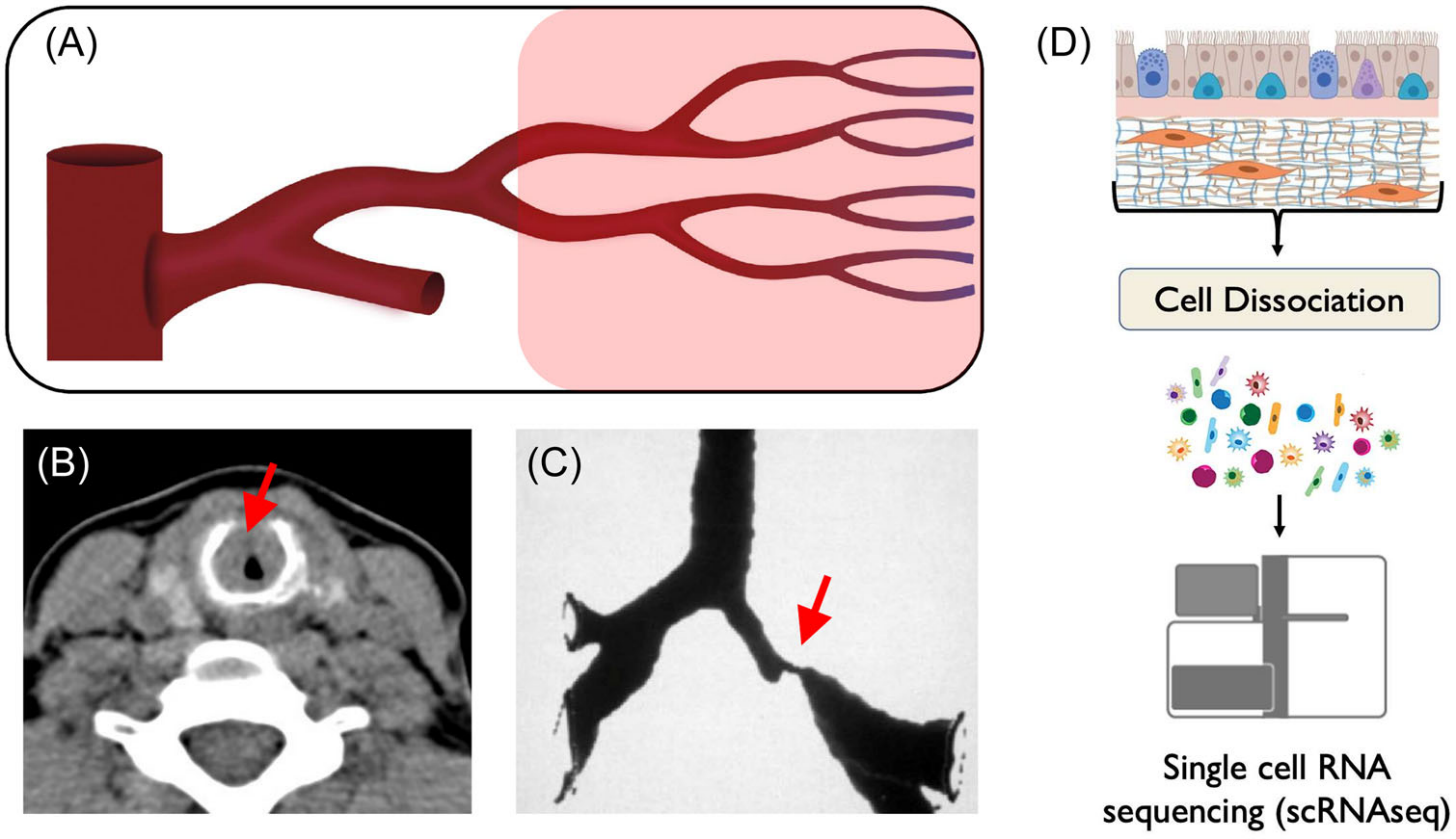
Schematic diagram for (A) GPA‐associated auto‐immune vasculitis with (B) subglottic and (C) bronchial sites of airway stenosis. (D) Pipeline for processing of airway biopsy specimens, cell dissociation and subsequent single‐cell RNA sequencing.

While progress has been made in the development of successful treatment regimens for systemic disease,^8^, ^11^ ameliorating airway disease in GPA remains a dramatic, unmet need.^12^ Despite significant survival gains over several decades, few patients with GPA emerge from a period of active disease without sustaining damage from the disease, its treatment, or both.^13^ Patients with GPA airway disease (subglottic stenosis and/or bronchial stenosis) report a significant decrement in their quality of life secondary to impaired activities of daily living and restricted communication.^14^ This often culminates in patients with GPA experiencing severe social, emotional, and work^15^ disability superimposed on an irreversible medical condition as they struggle to maintain their standard activities. Several studies have shown that the course of airway stenosis in GPA runs independently of the systemic disease,^16^, ^17^, ^18^ and is often refractory to standard systemic therapy.^19^ The role of current therapies in addressing the airway complications of GPA is not well defined and there is a lack of consensus regarding optimal airway treatment. Some authors suggest stenosis should be treated during active disease with dilatation and intralesional glucocorticoids.^20^ Other local interventions include dilatation,^21^ laser resection,^16^ or cryotherapy. Topical mitomycin C had been reported to reduce recurrence rates but now has fallen out of favor due to its toxicity. Stents are generally avoided because they contribute to persistent inflammation and disease reactivation.^22^ Open resection of the stenotic trachea and end‐to‐end tracheal repair has also been reported,^23^ along with augmentative cartilage laryngotracheoplasty^6^ but both are uncommonly performed. Despite these efforts nearly all patients with GPA‐associated airway stenosis will have recurrent stenosis by 5 years (49% at 1 year, 70% at 2 years and 80% at 5 years).^24^ More than 30% progress from their initial site of airway involvement,^25^, ^26^ and 20%–40% require a tracheostomy.^27^, ^28^

The factors responsible for the varied range of GPA disease manifestations and airway involvement are poorly understood at this time. As a result, investigation into both the identity and function of local cell populations in upper airway scar is a critical first step. Although limited prior studies have investigated GPA airway^29^ and mucosal^30^ biopsies using immunohistochemistry and flow cytometry, whole transcriptomic analysis at the single‐cell level has not yet been performed. Traditionally, bulk RNA‐sequencing had been used to evaluate gene expression in a sample population.^31^ However, these data reflect an averaged/pooled readout and are unable to resolve cell‐to‐cell variability in an otherwise heterogeneous cell population. Thus, single‐cell RNA sequencing (scRNAseq) provides the distinct advantage of interrogating gene expression patterns and molecular characteristics within each individual cell. This has been particularly useful in identifying inflammatory mediators in autoimmune rheumatologic processes, where there is significant heterogeneity in diseased tissues.^32^ In rheumatoid arthritis, for example, scRNAseq has allowed for the identification of synovial fibroblasts and macrophages as key players within the disease process, and these cells are currently being studied as therapeutic targets.^33^, ^34^

In this study, we aimed to characterize the immune landscape of GPA airway disease using single‐cell RNA sequencing (scRNAseq). Identifying effector populations that are involved in the pathogenesis and progression of airway stenosis in GPA will not only increase our understanding of the disease process at large, but also provide potential targets for the development of therapeutics to control airway disease (subglottic stenosis and bronchial stenosis).

## Materials and Methods

### Patients and Biopsy Acquisition

A total of 9 GPA, 7 idiopathic subglottic stenosis (iSGS), 5 post‐intubation proximal stenosis (PIPS), and 10 healthy control patients were included in this study. iSGS, PIPS, and GPA diagnoses were defined by established diagnostic criteria.^20^, ^35^ Biopsies of airway mucosa were obtained during airway endoscopic procedures. Three of the 10 healthy control samples were sourced from publicly available data, with tissue samples originating from tracheal tissue samples of recently deceased organ donors.^36^ The other seven healthy control samples were acquired during routine direct laryngoscopies for vocal fold injection augmentation. Two GPA patients had subglottic scar tissue biopsies collected at multiple time points (GPA patient 1: biopsy collection time points: *t*1 = 0 months, *t*2 = 7 months; GPA patient 2: biopsy collection: *t*1 = 0 months, *t*2 = 10 months, *t*3 = 13 months). Formal IRB approval was obtained for biopsy collection and analysis (Vanderbilt University Medical Center #140429). Consent was obtained from each patient.

### Single Cell RNA Sequencing

Tissue biopsies were processed using established protocols for scRNAseq **(** Figure 1D **)**. Briefly, 10× Genomics Chromium single‐cell immune profiling platform was performed in accordance with manufacturers' protocols. Suspended cells were loaded onto Chromium Next Gem Single Cell chip for cellular barcode addition, mRNA amplification, and subsequent RNA sequencing. Raw data was processed using the 10× Genomics Cell Ranger pipeline (v6.0.2) to produce scRNAseq data. Integration of single‐cell data across samples was conducted via Harmony^37^ and downstream processing was conducted using the Seurat package^38^, ^39^ in the R programming language (R Foundation for Statistical Computing). Filtration was performed to include cells possessing between 200 and 10,000 unique genes, greater than 500 unique read counts, and a maximum mitochondrial content of 20%. Unsupervised cell type identification was performed using Signac^39^ and SingleR^40^ with further manual validation using canonical markers for each cell type population.

### Genetic Analysis of GPA Risk Alleles

Following scRNAseq data preparation, 14 GPA risk alleles identified from prior genome‐wide association studies (GWAS)^41^, ^42^, ^43^ were further characterized in our samples. The average genetic expression level and percent of cells expressing each gene were quantified for each cell type using built‐in Seurat functions. The aggregate expression of the combined GWAS gene set was determined using modularity score analysis. The modularity score provides a relative expression level considering each gene supplied, which can be used to localize the overall expression of these genes to a specific group of cells.

### Pseudotime Trajectory Analysis

To investigate the dynamic changes within T cell populations, aggregate T cells from all patient samples were processed for standard trajectory analysis.^44^ The R package Monocle 3, version 1.3.1 was employed to perform trajectory inference on T cells with low‐dimensional representations from Seurat.^45^, ^46^ The Uniform Manifold Approximation and Projection (UMAP) embeddings and cell clusters obtained from Seurat served as input for Monocle 3, which performed trajectory graph learning and pseudo‐time measurement through reversed graph embedding.

### Data Visualization and Statistical Analysis

All GPA, iSGS, PIPS, and healthy control scRNAseq samples were plotted via UMAP dimensional reduction with cell type clusters identified using Signac and SingleR in R. Gene expression dot plots for GPA risk alleles were produced using Seurat package in R with percent of cells expressing (dot size) and average level of expression (dot color) quantified for each gene and cell type. Bar graphs were created using Prism version 9.0 (GraphPad Software Inc.). Pseudotime trajectory analysis was performed with Monocle 3 package in R with the start time placed centrally at the CD4+ effector population. Statistical testing was also performed using Prism with an *α* value of .05.

## Results

### Cellular Profiling of Airway Scar in GPA and Healthy Mucosa

**Figure 2:**
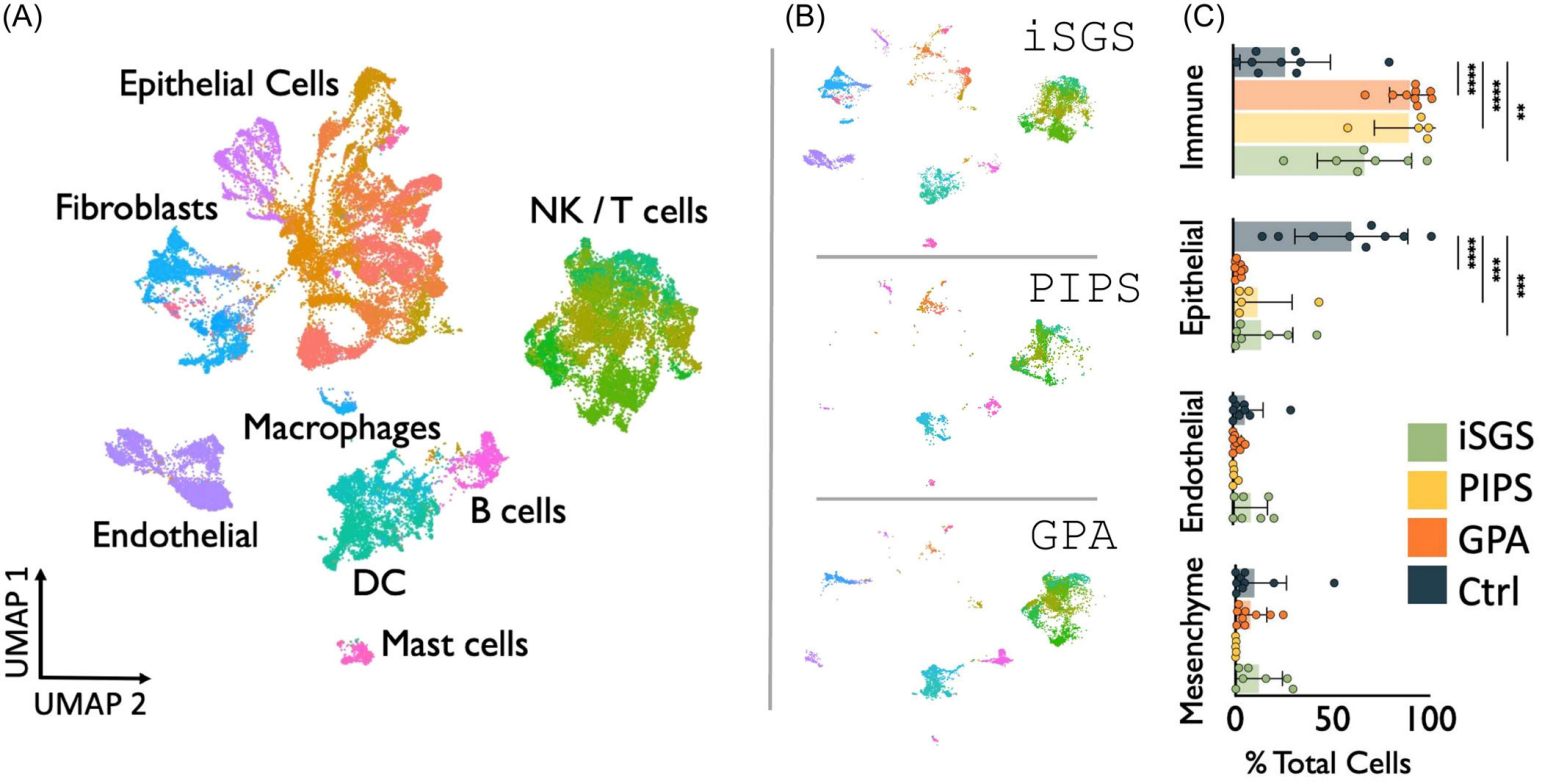
iSGS, PIPS, GPA, and control scRNAseq samples were plotted via UMAP dimensional reduction into (A) specific cell types and (B) parsed into disease comparators. (C) Various cell populations were compared between disease and control states.

**Figure 3:**
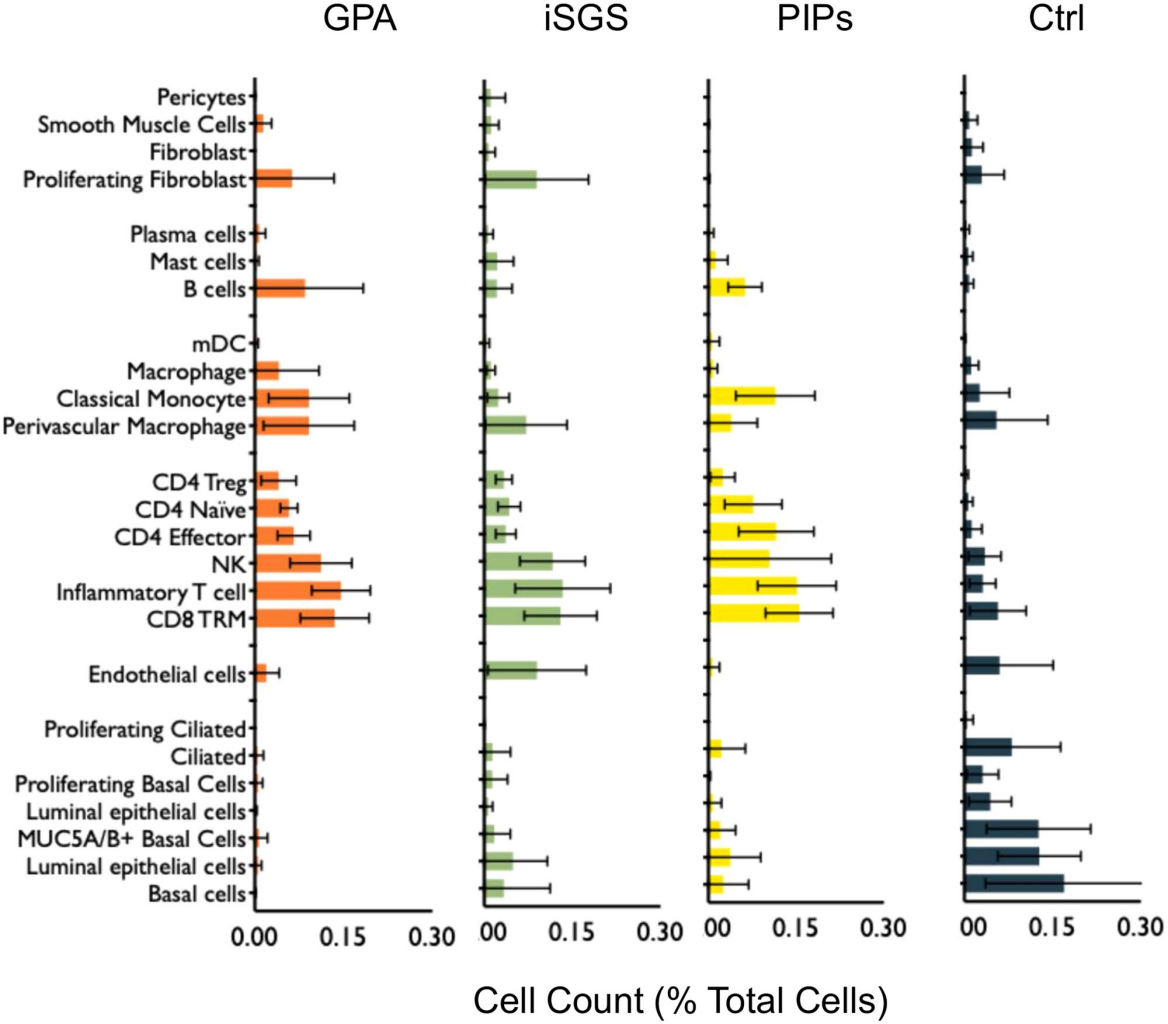
Quantification of immune, epithelial, endothelial, and mesenchymal cell populations was performed and compared between disease and control states. Data are shown as cell count as percentage of total cells.

We aimed to first characterize the distribution of cellular populations in GPA airway scar as compared to iSGS, PIPS and normal mucosal controls. Using scRNAseq, distinct clustered cell populations were represented using UMAP visualization of all single cell data from GPA, iSGS, PIPS, and normal samples (Figure 2A), with further delineation into iSGS, PIPS, and GPA samples alone (Figure 2B). Similar to airway scar from patients with iSGS and PIPS, GPA airway scar demonstrated significantly increased populations of immune cells when compared to mucosal controls (Figure 2C). In contrast, there was an observed decrease in epithelial cells in iSGS, PIPS and GPA airway scar when compared to controls. No differential population shifts were observed in endothelial or mesenchymal cells across all samples. Further characterization of specific cell populations (eg, macrophage, CD4 naïve, basal cells, etc) was performed and depicted by cell counts as percent of total cells (Figure 3). These results demonstrate that like patients with iSGS and PIPS, patients with GPA exhibit robust populations of infiltrating immune cells in diseased airway tissue.

### GPA Risk Alleles Are Primarily Expressed in Immune Cell Populations

**Figure 4:**
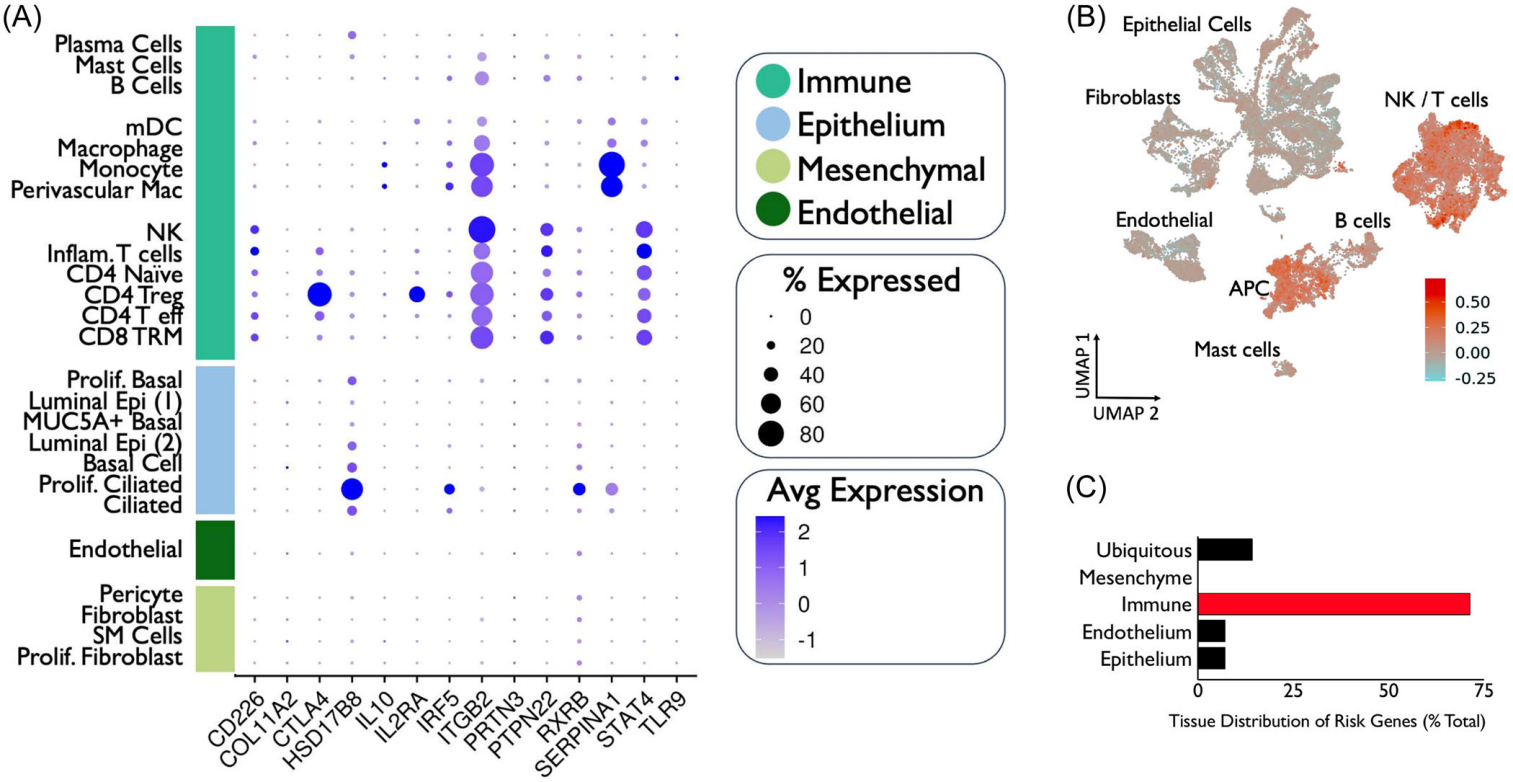
(A) Expression of established GPA risk alleles was analyzed across cell types. (B) Heat map expression of GPA risk alleles superimposed on scRNAseq UMAP. (C) Quantification of genetic distribution of risk genes by cell type.

To further classify the localization of GPA‐related genes, we performed differential gene expression on established candidate GPA risk alleles^41^, ^42^, ^43^ across cell lineages in our scRNAseq data. The 14 established GPA risk alleles were primarily expressed in immune cell populations (Figure 4,B). Additional quantification of tissue distribution revealed that 60% of risk alleles were primarily expressed in immune subsets as compared to <20% ubiquitously expressed and <10% each expressed in endothelial and epithelial cells (Figure 4C). Furthermore, when specifically parsing out immune cell subsets, 10 (71%) GPA risk alleles localized to T cells and macrophages.

### An Observed Subset of Inflammatory T Cells Are Unique to GPA Patients

**Figure 5:**
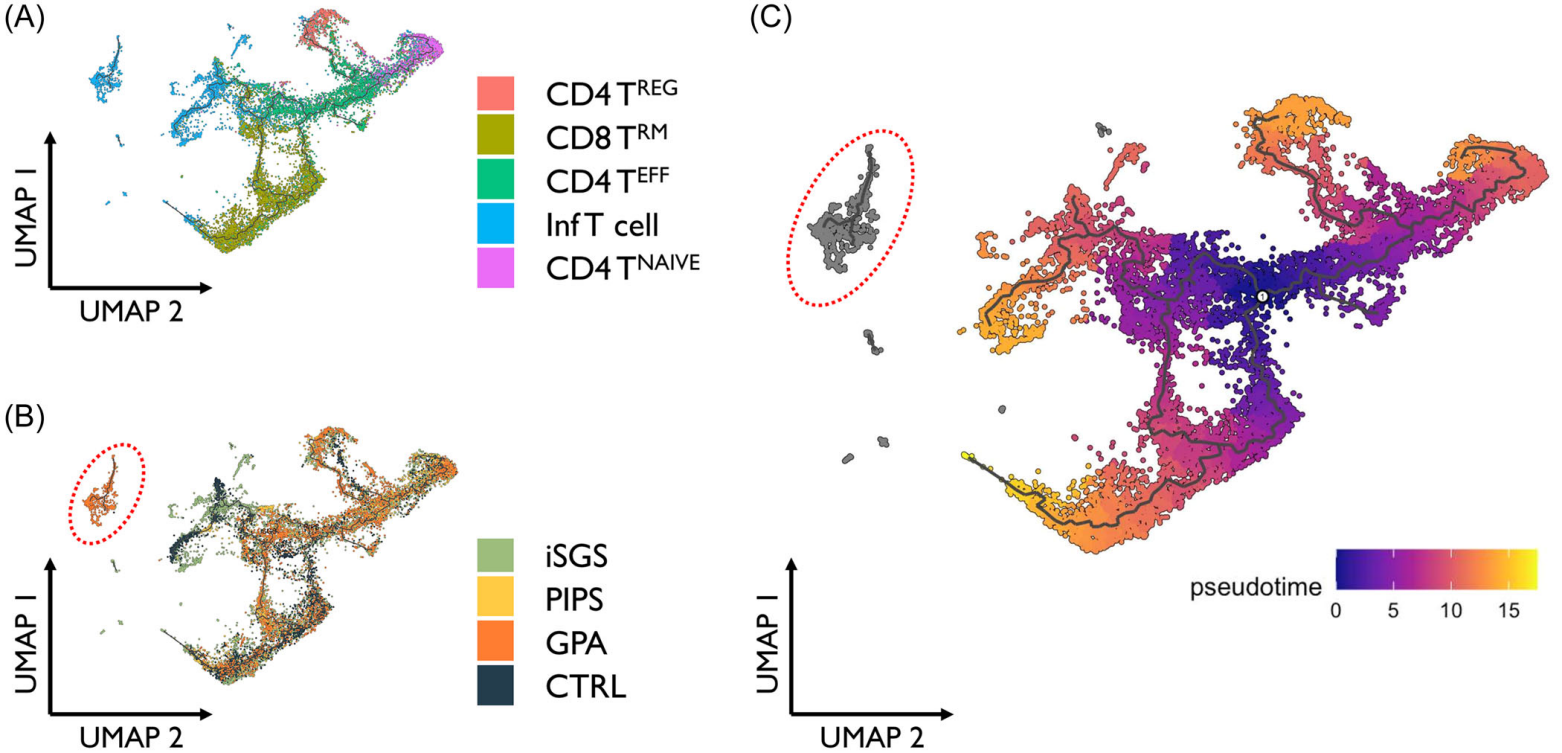
(A) Visualization of T cells from all scRNAseq samples (B) T cells labeled by disease with identification of T cell subset unique to GPA. (C) Pseudotime analysis with start time at the CD4+ effector population.

Given the observed expression of GPA risk alleles in immune cell subsets, particularly in T cells, we sought to compare T cell populations across disease states. UMAP visualization of single‐cell sequencing data of T cell subsets across all patient samples demonstrates robust populations of CD4^+^, CD8^+^, and inflammatory T cells (Figure 5A). Further delineation into disease states (iSGS, PIPS, GPA, control) reveals a sub‐population of inflammatory T cells that almost exclusively originates from GPA samples (Figure 5B). To further characterize this subset and evaluate cell trajectory and lineage, pseudotime analysis was performed using Monocle 3.^45^, ^46^ Placing the start time centrally within the CD4^+^ effector T cell population, three distinct trajectory branches were observed (Figure 5C). However, the observed GPA‐specific inflammatory T cell subset remained distinct and separate from the rest of the T cell population.

Additional differential gene expression analysis was also performed comparing inflammatory T cell populations (Supplemental Figure 1, available online). When comparing GPA to normal, there were 18 significant differentially expressed genes (16 downregulated, 2 upregulated). GPA, compared to iSGS, had 936 significant differentially expressed genes (277 downregulated, 659 upregulated), and when compared to PIPS, had 910 significant differentially expressed genes (244 downregulated, 666 upregulated).

## Discussion

The pathogenesis and progression of GPA airway disease is poorly understood and historical treatment algorithms have had varying levels of success. Although autoimmunity and immune‐related pathways have previously been implicated in disease,^5^, ^47^, ^48^ to our knowledge, single‐cell characterization of the infiltrating immune cells in GPA airway scar has not been previously done. In this study, we provide new data on immune cell composition in GPA airway disease and identify a subset of T cells that is unique to GPA scar.

We found that GPA airway scar exhibits a robust population of infiltrating immune cells, with more immune cells and less epithelial cells compared to normal mucosal controls. Furthermore, when characterizing tissue localization of established candidate GPA risk alleles,^41^, ^42^, ^43^ a majority were expressed among immune cell populations. Similar to other inflammatory pulmonary disease states,^49^ these data suggest that immune‐mediated airway inflammation and fibrosis^50^ also play a pivotal role in local GPA airway disease. Although disease pathogenesis/progression is not currently well‐described, auto‐immune dysregulation has classically been implicated in GPA, with anti‐neutrophil cytoplasmic auto‐antibodies (ANCA) against serum protease proteinase 3 (PR3) present in 80‐90% of cases.^51^ More recent data have also demonstrated an association between PR3 and the activation of plasmacytoid dendritic cells and polarization of Th2, Th9, and Th17 cells.^52^ Our results confirm the presence of a significant immune infiltration in GPA airway scar and also suggest functional changes in immune dysregulation with expression of established genetic risk variants demonstrating a predilection for immune cell populations.

In addition, we found that a large majority of GPA risk alleles localized to T cells and macrophages, suggesting key roles for these cell populations in disease activity. This is in concordance with prior data investigating respiratory biopsies in GPA patients with active airway disease.^29^ In this study, Silva de Souza et al. found significantly higher percentages of macrophages and T cells among 35 GPA patient biopsies. To further delineate effector immune populations, we parsed out specific T cells across all patient samples and performed differential gene expression analysis. Interestingly, in CD4^−^/CD8^−^ inflammatory T cells specifically, we observed significant gene expression changes when GPA was compared to iSGS and also when GPA was compared to PIPS. On the other hand, when GPA was compared to control samples, there were minimal gene expression differences between these two groups within the CD4^−^/CD8^−^ inflammatory T cell population. These findings suggest that the inflammatory T cell population as a whole appears to be more functionally similar between GPA and control groups as opposed to those from iSGS and PIPS. However, when further classifying by disease state, a subset of these inflammatory T cells almost exclusively originated from GPA patients. Using Monocle 3 to evaluate single‐cell trajectories based on predicted gene expression changes,^44^, ^45^ our results also demonstrate that this population of inflammatory T cells appears to be distinct and separate from other T cell populations as well. Taken together, our findings indicate that GPA airway scar possesses a unique immune signature within inflammatory T cells that may contribute to the observed differences in disease biology.

There are some limitations that are present in this study, particularly with regard to single‐cell RNA sequencing. Given the resource intensive nature of scRNAseq as well as the rarity of GPA, our sample size was limited. In addition to this, sequencing data only presents data at a discrete time point and lacks information on changes over time. To approximate this, we utilized pseudotime analysis, which has been previously used to study temporal relationships in single cell data including cell cycle,^53^ lineage‐specific gene regulation,^54^ and pluripotent stem cell differentiation pathways.^55^ However, to validate our findings, additional functional and sequencing experiments at multiple time points (eg, active disease and remission) may be needed.

This study characterizes, for the first time, the local immune cell populations in GPA airway scar as well as the associated expression of GPA risk alleles. It also identifies the presence of a unique GPA‐specific population of inflammatory T cells. These novel findings highlight both the overall GPA airway immune landscape as well as a potential effector population of immune cells that play a role in GPA‐associated airway stenosis. At this time, additional functional studies are needed to further substantiate the role of these inflammatory T cells in disease pathogenesis and progression. Furthermore, other avenues of study may include interrogating the airway microbiome in this patient population and its interplay with airway immune cells. As prior studies have demonstrated a link between *Staphylococcus aureus* colonization and GPA disease relapse,^56^ establishing a mechanistic pathway in this area could yield a number of therapeutic targets. Potential local and systemic treatments could involve anti‐microbial agents, T cell depleting therapies, immune modulators, and more. Finally, given that mechanisms such as defects/alterations in respiratory epithelium,^57^, ^58^ pathogen exposure and translocation,^59^ and subsequent fibrotic scar creation have been demonstrated in multiple other airway corollaries, improving our understanding in GPA may provide further insights into other diseases with airway inflammation/stenosis as well.

## Author Contributions

**Wenda Ye**, conceptualization, formal analysis, original draft preparation, review and editing; **Evan Clark**, formal analysis, review and editing; **Edward Talatala**, review and editing; **Ruth Davis**, conceptualization, review and editing; **Marisol Ramirez‐Solano**, data curation, formal analysis; **Quanhu Sheng**, data curation, formal analysis; **Jing Yang**, data curation, formal analysis, **Sam Collins**, supervision; **Alexander Hillel**, supervision; **Alexander Gelbard**, conceptualization, formal analysis, supervision, review and editing.

## Disclosures

### Competing interests

None.

### Funding source

AAO‐HNS CORE Grants Program, Vanderbilt SCRIPS Pilot Grant, Vanderbilt VICTR Institutional Grant.

## Supporting information

Supplementary Figure 1. Differential gene expression performed comparing inflammatory T cells across disease comparators (iSGS, PIPS, GPA and control).
