# Supplementary figures and images for: Characterizing the Cellular Constituents of Proximal Airway Disease in Granulomatosis With Polyangiitis
Source: Otolaryngol Head Neck Surg. 2025 Mar 10;172(6):2009–17. doi: 10.1002/ohn.1197 (PMC12120036; doi:10.1002/ohn.1197)

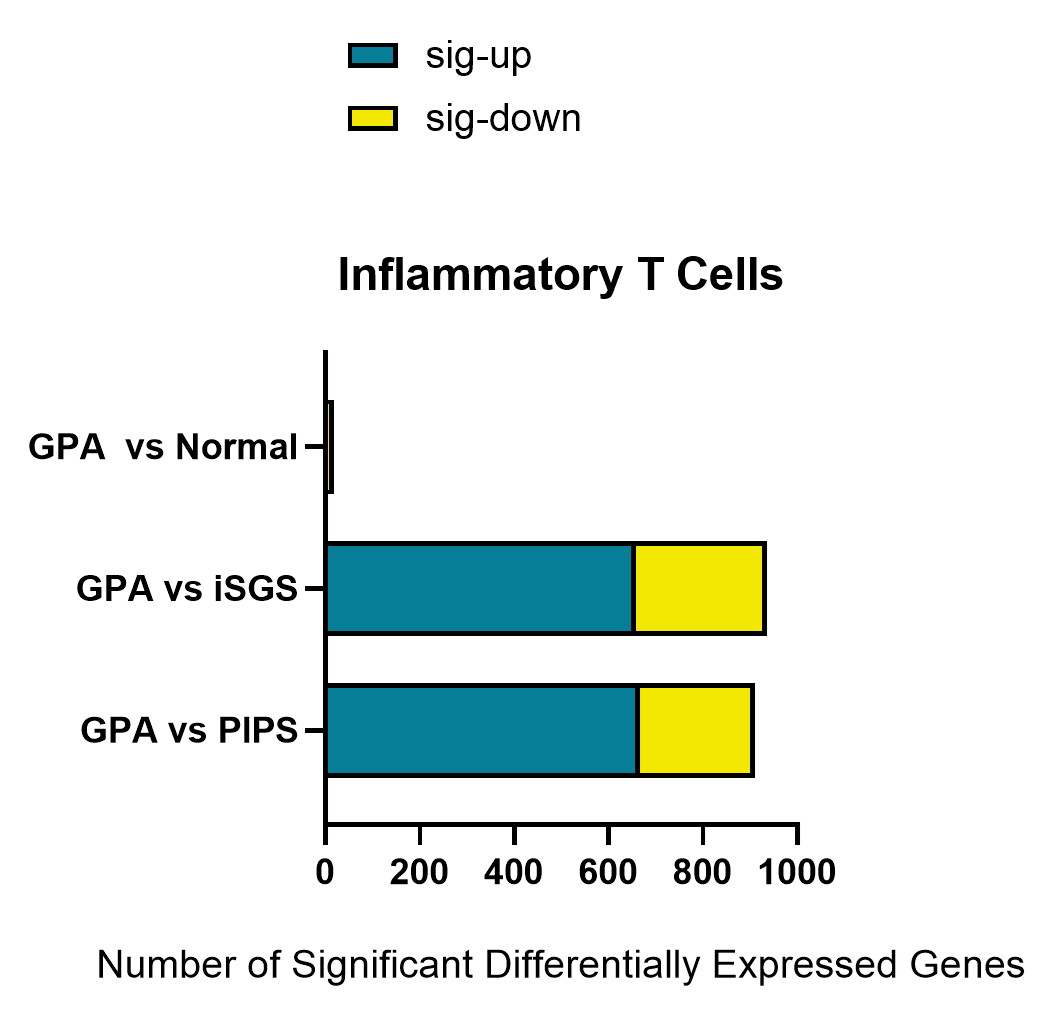

Supplement: Supplementary file 1 — Supplementary Figure 1. Differential gene expression performed comparing inflammatory T cells across disease comparators (iSGS, PIPS, GPA and control). [file OHN-172-2009-s001.png]
